# Matrix Metalloproteinase-2 Polymorphisms and Incident Coronary Artery Disease: A Meta-Analysis

**DOI:** 10.1097/MD.0000000000000824

**Published:** 2015-07-13

**Authors:** Yujie Shi, Jian Zhang, Chen Tan, Wei Xu, Qi Sun, Junxia Li

**Affiliations:** From the Cardiovascular Diseases Institute, General Hospital of Beijing Military Command of PLA, Beijing, China.

## Abstract

Previous studies have yielded controversial results related to the contribution of matrix metalloproteinase-2 (MMP-2) -1306 C/T and -735 C/T polymorphisms in the progression of coronary artery disease (CAD). This study aimed to provide strong evidence for the role of the 2 polymorphisms in genetic risk of CAD.

The human case-control studies regarding the association of MMP-2 polymorphisms with CAD risk were systematically identified through online databases (PubMed, Embase, the Cochrane Library, and CNKI) and manual search. Inclusion criteria were defined for the eligible studies. The fixed-effects meta-analysis was performed to combine the values when homogeneity was indicated. Alternatively, the random-effects meta-analysis was utilized.

A total of 2118 samples were analyzed in the meta-analysis of -1306 C/T. The odds ratio for the initially tested genetic model was 0.93 (95% confidence interval: 0.78–1.10 under TT + CT vs CC). The remaining comparisons similarly showed -1306 C/T genotypes were not significantly associated with the risk of CAD. We noted the same trend when data were retrained to myocardial infarction studies. Meta-analysis of -735 C/T suggested no clear association with the development of CAD.

The results of the current work fail to support a significant involvement of MMP-2 -1306 C/T and -735 C/T polymorphisms in the risk of developing CAD.

## INTRODUCTION

The reference of family history to coronary artery disease (CAD) was first identified by a British investigator in mid-1960s.^1^ Later a long list of twin studies and family aggregation studies aiming to validate the role of family factors in incident CAD have lent support to the earliest report,^2–5^ with a most recent research article suggesting individuals who have 2 or more affected relatives, compared to the individuals without any family history, are more likely to develop CAD.^6^ The human genetic evidence has intensified interest in epidemiological studies of inheritance contribution and the development of this common cardiovascular system disease.

Many well-recognized pathways are essential components in the progression and complications of CAD, including matrix metalloproteinase (MMP). Activation of MMP is believed to accelerate plaque rupture and thrombus formation by impairing the fibrous cap and degrading extracellular matrix proteins.^7^ Higher levels of MMP induced by inflammation have been reported to cause plaque breakage and pathological matrix destruction; promoting MMP down-regulation or blocking their activities is an effective way to prevent the formation of CAD.^8^

Matrix metalloproteinase-2 (MMP-2) is an important member of MMP family involved in several cellular and natural processes (cell development, transfer, healing of injured tissue, and scar formation), and functions as a proteolytic enzyme catalyzing matrix proteins, basement membrane constituents in particular.^9^ The polymorphic MMP-2 is located at chromosome 16q13 with 13 exons within its promoter region. MMP-2 single nucleotide polymorphisms (SNPs) enable alterations in transcriptional regulation and thereby affect the function of its enzyme.^10^ These functional SNPs also affect MMP-2 gene transcription in an allele-specific fashion and are thus considered potential candidate to evaluate the associations with atherogenesis, tumor initiation, invasion, and metastasis.^11^

There have been many polymorphisms widely studied in CAD community, including MMP-2 -1306 C/T and -735 C/T promoter SNPs. A notably decreased or increased enzyme activity has been linked to the -1306 T allele and the C allele, respectively.^11,12^ Most strikingly, the -735 T allele even leads to elimination of the promoter activities.^13^ Due to the functional importance, several groups have associated the 2 polymorphisms with incident CAD, a heterogeneous and common vascular disorder that has caused a large number of deaths worldwide.^6^ The published data nevertheless fail to yield a consistent conclusion most likely because of the inadequate sample size.^14,15^ Herein, we aimed to determine whether or not the MMP-2 SNPs act as modifiable risk factors for CAD by use of a meta-analysis.

## METHODS

### Ethical Review

The study was approved by the ethics committee of general hospital of Beijing military command of PLA.

### Literature Search

Online databases (PubMed, Embase, the Cochrane Library, and CNKI) were used to seek the studies of the association between MMP-2 polymorphisms and CAD risk, without language restriction. The MeSH search terms and keywords included coronary artery disease, matrix metalloproteinase-2, polymorphism, polymorphisms, and their synonyms (eg, coronary heart disease, myocardial infarction, gelatinase A, variant, variants) and abbreviations. Additional data were obtained by scanning the reference lists of review articles and research articles that fulfilled all of the following inclusion criteria:

First, the study must be published before the literature search was terminated (December 2013);

Second, CAD or MI patients must be investigated and the control group comprised healthy individuals rather than non-CAD or non-MI patients to guarantee the accuracy of estimations;

Third, at least 1 of the MMP-2 polymorphisms, either -1306 C/T or -735 C/T, was studied;

Fourth, the original article must report ample genetic data by which the risk of CAD could be estimated;

Fifth, there was no notable deviation from Hardy–Weinberg equilibrium (HWE) in control group.

Any study violating the predesigned criteria was not considered in this meta-analysis.

### Data Abstraction

To minimize the possibility of biased results induced by erroneous data abstraction, 2 investigators independently extracted authors, country of origin, ethnicity, publication year, matching status, adjusted factors, type of disease (CAD or MI), and genotype frequency, using a standardized protocol. Discrepancies were resolved through discussion with the third investigator.

### Statistical Analysis

Crude odds ratios and its 95% confidence intervals were pooled to evaluate the correlation between MMP-2 polymorphisms and CAD risk. We first used TT + CT versus CC genetic comparison, then TT versus CT + CC, TT versus CC, and CT versus CC. Pooled ORs were combined using the fixed-effects meta-analysis in the absence of substantial heterogeneity^16^; conversely, the random-effects meta-analysis was employed.^17^ The degree of heterogeneity across studies was evaluated using the I2 statistic,^18^ with I2 >50% being considered large heterogeneity. In order to check if the combined estimates were stable, more specifically, whether the single studies influenced the results materially, we performed sensitivity analysis. HWE in controls was determined using the χ2 test. Both the funnel plots and Egger linear regression test were used to evaluate the publication bias in this meta-analysis.^19^

All statistical data were analyzed via the Stata software (version 12.0; StataCorp LP, College Station, TX). The significance threshold was set at P values below .05.

## RESULTS

### Literature Selection and Characteristics

**FIGURE 1:**
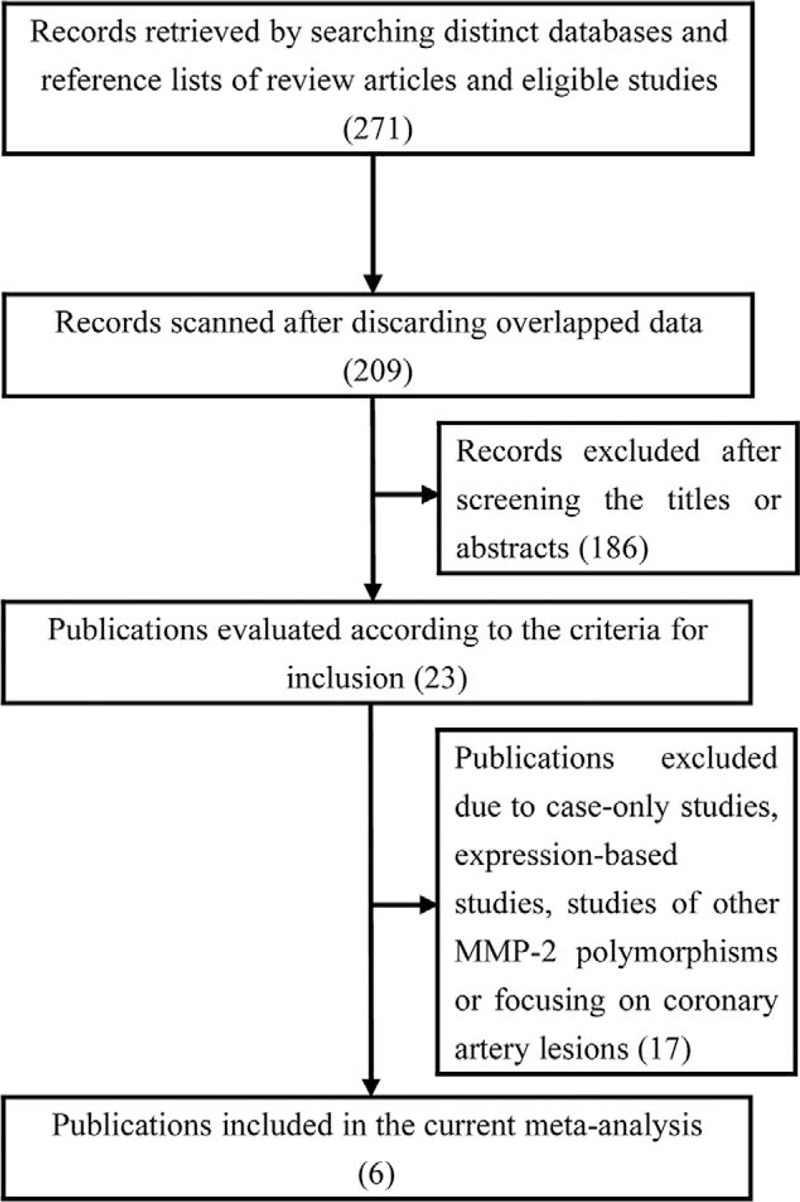
The flow chart of included studies in the meta-analysis.

Online literature search supplemented manual search resulted in 271 records. We initially excluded 62 overlapped records and subsequently discarded 186 publications after screening the titles or abstracts. We then evaluated the eligibility for inclusion for the remainders and finally excluded 17 publications after detailed check (11 expression-based studies, 3 case-only studies, 2 studies of other MMP-2 polymorphisms, 1 study focusing on coronary artery lesions). We therefore identified 6 eligible studies for this analysis^14,15,20–23^ (see Figure 1).

As displayed in Table 1, there were 5 studies for MMP-2 -1306 C/T, with 2 studies of Mexican samples, 2 of Caucasian samples, and 1 of East Asian samples. The matching status varied substantially across the studies, including 2 publications not reporting required information. This substantial variance was also seen in adjusted factors. In terms of -735 C/T, a total of 3 studies were analyzed in the final analysis. East Asian, Caucasian, and Mexican populations were involved and the matching as well as the adjustment confounders were not uniformly defined. None of the studies showed significant departure from HWE (P > .05).

**TABLE 1:**
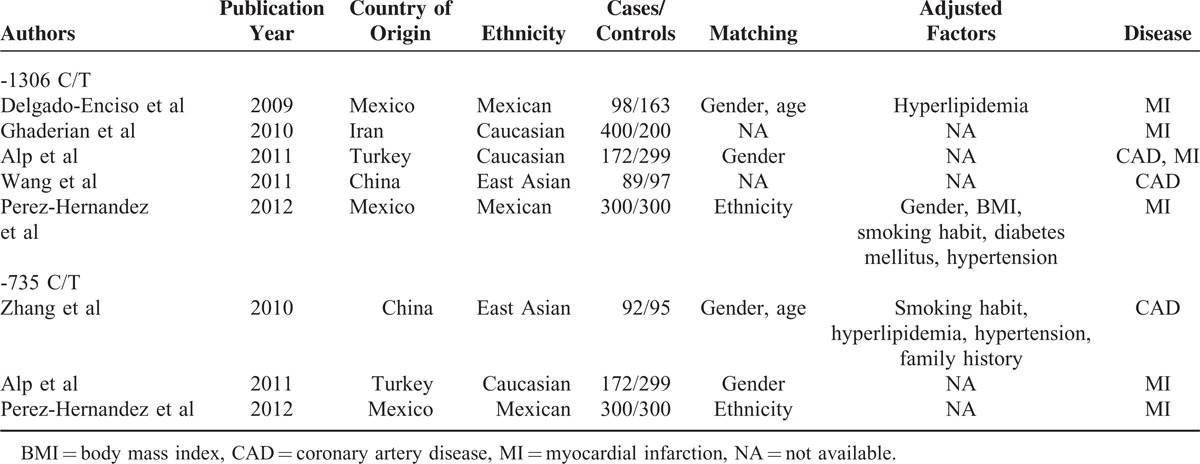
Main Characteristics of Eligible Studies

### Association Between MMP-2 Polymorphisms and CAD Risk

**FIGURE 2:**
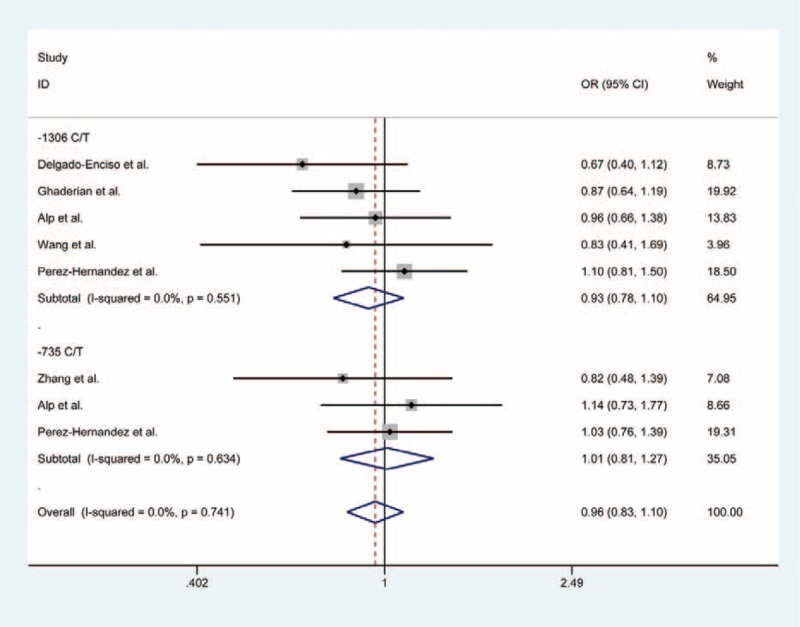
Forest plot of overall CAD risk associated with MMP-2 polymorphisms under TT + CT versus CC. Compared to CC genotype, TT + CT genotypes showed no increased risk of CAD. Fixed-effects meta-analysis was performed. CAD = coronary artery disease, CI = confidence interval, MMP-2 = matrix metalloproteinase-2, OR = odds ratio.

**FIGURE 3:**
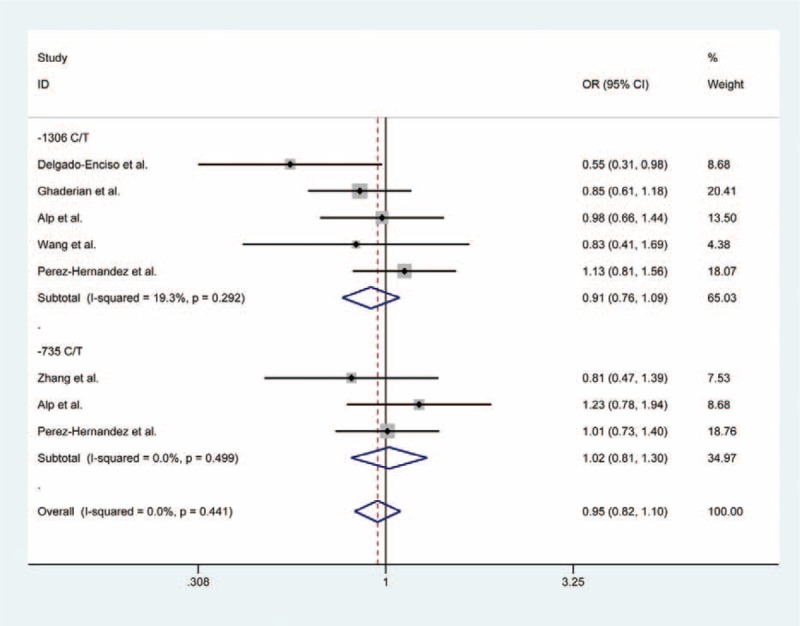
Forest plot of overall CAD risk associated with MMP-2 polymorphisms under CT versus CC. Compared to CC genotype, CT genotype showed no increased risk of CAD. Fixed-effects meta-analysis was performed. CAD = coronary artery disease, CI = confidence interval, MMP-2 = matrix metalloproteinase-2, OR = odds ratio.

Fixed-effects meta-analysis of 1059 CAD cases and 1059 healthy controls suggested that individuals carrying the TT + CT genotypes of MMP-2 -1306 C/T did not have higher or lower risk of CAD (odds ratio: 0.93, 95% confidence interval: 0.78–1.10 under TT + CT vs CC) (see Figure 2). Neither did we find any significant association when assuming TT versus CT + CC (odds ratio: 1.05, 95% confidence interval: 0.69–1.62), TT versus CC (odds ratio: 1.00, 95% confidence interval: 0.65–1.55), and CT versus CC (odds ratio: 0.91, 95% confidence interval: 0.76–1.09) (see Figure 3). For the association of -1306 C/T and MI risk, 1904 samples were analyzed. As shown in Table 2, -1306 C/T genotypes were not significantly associated with risk of MI.

**TABLE 2:**
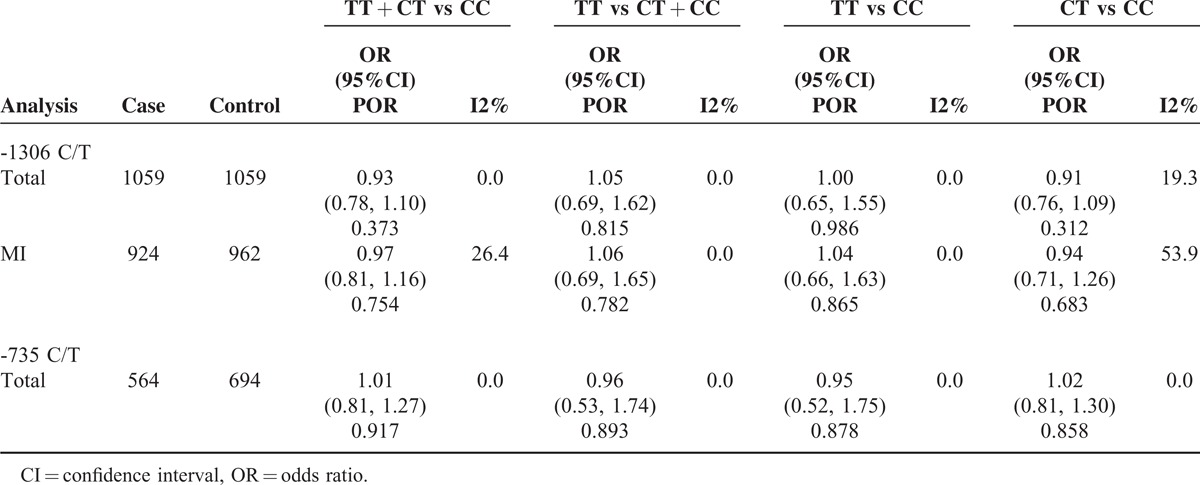
Meta-Analysis of the Association Between MMP-2 Polymorphisms and CAD

Results of the overall meta-analysis for the association between -735 C/T and CAD risk are shown in Figures 2 and 3, and Table 2. The genetic comparisons tested revealed a nonsignificant association with CAD risk. All I2 statistics were less than 50%, suggesting our findings were not quantitatively affected as a result of interstudy heterogeneity.

### Sensitivity Analysis

To test the stability of the combined results, we performed sensitivity analysis via sequentially removing the independent studies. No notable alternation was seen throughout the leave-one-out analysis, and this procedure ensured the stability and credibility of the data in this meta-analysis (data not shown).

### Publication Bias

**FIGURE 4:**
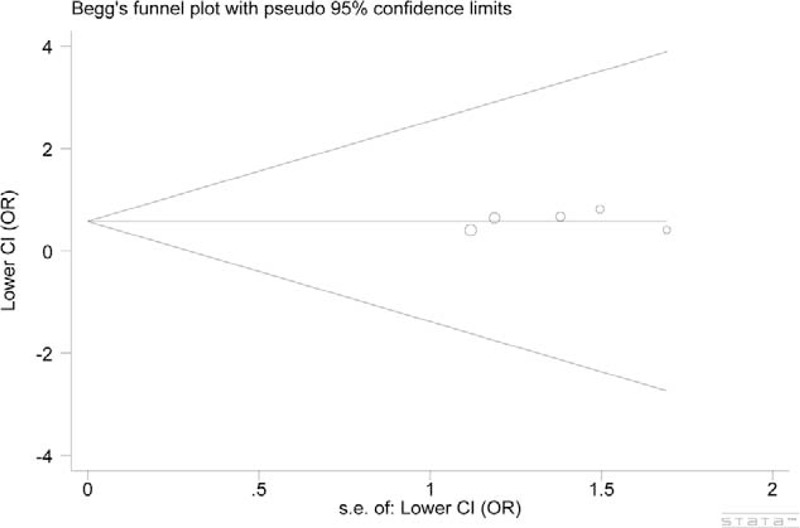
Funnel plot analysis to detect publication bias across the literature of MMP-2 -1306 C/T (TT + CT vs CC). Each point represents a separate study for the indicated association. CI = confidence interval, MMP-2 = matrix metalloproteinase-2, OR = odds ratio.

We used Begg funnel plot and Egger test to evaluate the publication bias. The studies of MMP-2 polymorphisms were symmetrically distributed within the funnel plots and the Egger test provided statistical evidence of little publication bias across the literature (-1306 C/T: PBegg = 0.462, PEgger = 0.676; -735 C/T: PBegg = 0.308, PEgger = 0.158; TT + CT vs CC) (Figure 4).

## DISCUSSION

CAD represents a major type of heart disease and has been a global health problem bedevilling an increasing number of people.^24^ Although external factors such as changes in dietary patterns, an increased prevalence of smoking, and intense pressure are taken as contributory factors for the development of CAD, the research focus in recent years has shifted to the genetic contributions to this invasive heart disease. On the basis of 1059 patients with CAD and 1059 control subjects, we estimated the relationship of MMP-2 polymorphisms with CAD susceptibility by means of meta-analysis, a widely used quantitative approach that could provide strong evidence for SNP-malignancy associations. We found -1306 C/T genotypes were not significantly associated with CAD risk using the genetic models listed in Table 2. Subsequent analysis of MI studies showed similar nonsignificant associations. In the meta-analysis of data for -735 C/T, we observed the same trend as in the analyses of the former MMP-2 promoter polymorphism. Obvious publication bias or interstudy heterogeneity was not indicated by using corresponding tests.

Earlier studies have established the contribution of MMP activity to angiopathy initiation by transferring vascular smooth muscle cells into the intimal space which provides microenvironment for cellular proliferation and plaque formation via the internal elastic lamina.^22,25,26^ A recent study has associated atherosclerotic plaques with enhanced MMP expression levels.^27^ Downregulation of MMP-2 expression has been reported in both acute and chronic phases of coronary disease in vitro.^28^ Available data also demonstrated elevated circulating levels of MMP-2 in patients with acute coronary syndrome compared to those with stable effort angina.^29,30^ In patients with acute MI, Hojo et al observed a gradually increased plasma MMP-2 levels and the increase reached to the maximum on day 21 after onset.^31^ According to the previous data, we can speculate that the polymorphisms, the commonest variations in human genomics, in the promoter region of predisposition genes, such as MMP-2, probably act as modifiable risk factors for the development and progression of CAD. This speculation fails to lend support to the findings in the current work, where we found no association of MMP-2 polymorphisms and CAD incidence. There are several possible reasons for the inconsistency. The MMP-2 promoter polymorphisms as low-penetrance sequence variations require a large-scale study to detect their slight or moderate effects on CAD risk. Further, the different methodologies applied in each of the published studies may decrease the precision of genotyping and hence leads to biased results in the single studies and this meta-analysis consequently.

MMP-2 polymorphisms are of peculiar interest due to the functionally important role played in transcriptional activation activity of MMP-2 protein, including -1306 C/T and -735 C/T. A study of Mexican samples showed 2.05-fold increased risk of MI attributable to the -1306 CC genotype and this increase seemed more pronounced in obese or hypertensive subjects.14 It is interesting that a most recent study, also in a Mexican population, reported MMP-2 -1575G/A polymorphism, but not -1306 C/T and -735 C/T, was an independent risk factor involved in the risk of developing MI.^22^ Replication efforts for the association included Ghaderian et al, who found no statistically significant association between genetic polymorphisms of MMP-2 and risk of MI.^15^ No significant associations were later reported in a Turkish study.^20^ With respect to the 2 Chinese studies, one is for -1306 C/T and the other for -735 C/T, both of which failed to identify any significant relation with CAD.^21,23^ As most of the previously published studies have shown no involvement of the MMP-2 polymorphisms in modifying the risk of CAD or MI, we can infer that the genetic variants of interest are unlikely to play a major role in the disease concerned in this study.

In order to facilitate a better understanding of the effect estimations, several limitations of this analysis should be noted. First, our meta-analysis incorporated a limited number of studies, with each representing a small or moderate sample size, and this may result in false negatives as reported in the present work. Second, it is still unclear whether or not the MMP-2 polymorphisms modulate the risk of CAD or MI when data are restrained to some specific population, such as Mexican, Caucasian, and East Asian. Third, due to the considerable difference in adjustment confounders across the literature, we cannot evaluate the risk of CAD using an adjusted odds ratio.

Nevertheless, we have directed much effort to literature search and combined all data published to date, suggesting the first meta-analysis of MMP-2 -1306 C/T and -735 C/T SNPs has provided the strongest evidence of the genetic associations than any previous study. Therefore, our results are relatively robust and may have some implications for future investigations.

In conclusion, current evidence failed to support a significant association between -1306 C/T and -735 C/T polymorphisms at MMP-2 locus and the risk of CAD. Further research is clearly needed to determine the role of the 2 polymorphisms in the development of CAD and thus provides novel insights into the molecular mechanism that underlies genetic risk of the pervasive disease.
